# Case Report: First Report of T-Cell Large Granular Lymphocytic Leukemia With NPL-DHX9 Gene Fusion Successfully Treated With Cladribine: Clinical Experience and Literature Review

**DOI:** 10.3389/fonc.2022.824393

**Published:** 2022-05-06

**Authors:** Qin Hu, Yunfei Li, Ying Zhang, Shusen Sun, Hui Wang, Zhiping Jiang, Sheng Deng

**Affiliations:** ^1^ Department of Pharmacy, Xiangya Hospital, Central South University, Changsha, China; ^2^ National Clinical Research Center for Geriatric Disorders, Xiangya Hospital, Central South University, Changsha, China; ^3^ Institute of Hospital Management, Central South University, Changsha, China; ^4^ Department of Hematology, Xiangya Hospital, Central South University, Changsha, China; ^5^ Department of Pharmacy Practice, College of Pharmacy and Health Sciences, Western New England University, Springfeld, MA, United States; ^6^ Department of Pathology and Laboratory Medicine, Hebei Yanda Lu Daopei Hospital, Langfang, China

**Keywords:** large granular lymphocyte leukemia, invasive pulmonary aspergillosis, agranulocytosis with fever, NPL-DHX9, cladribine

## Abstract

**Background:**

T-cell large granular lymphocytic leukemia (T-LGLL) is a rare lymphoproliferative disorder that starts in T cells and is usually indolent. Long-term use of immunosuppressants, combined with agranulocytosis, is a double-edged sword, as both can lead to serious infections, especially in patients with combined hematologic malignancies and immune defects.

**Case Presentation:**

A 30-year-old female patient was admitted to the hospital because of agranulocytosis for five years, with chest tightness, fatigue, and fever for two days. Pathology and metagenomic next-generation sequencing (mNGS) detected Aspergillus. Although she received cyclosporine and methylprednisolone, the patient showed drug intolerance and progression with invasive pulmonary fungal infections. After a bone marrow aspiration biopsy and other related examinations, she was diagnosed with T-LGLL and invasive pulmonary aspergillosis (IPA). T-cell immunophenotype was CD45+CD3dim+CD5-CD4-CD8+CD7+CD57p+CD25-CD30-, TCRγδ+, transducer and activator of transcripton-3 (STAT3) Y640F mutation and fusion gene NPL-DHX9 rearrangement were confirmed, which has never been reported in hematological diseases. After voriconazole regimen adjustment during treatment based on therapeutic drug concentration monitoring (TDM) and improvement in lung infection, the patient finally treated with purine nucleoside analogues (PNA) cladribine as a single agent at 0.14 mg/kg/d for 5 days. Complete response was achieved after four-cycles cladribine treatment (WBC 2.1*109/L, HGB 117 g/L, PLT 196*109/L, ANC 1.6*109/L, and ALC 0.2*109/L).

**Conclusions:**

To our knowledge, this is the first case of T-LGLL with a rare γδ type and fusion gene NPL-DHX9 rearrangement. The patient was successfully treated with cladribine, suggesting that this regimen could be a promising therapeutic strategy for patients with aggressive T-LGLL.

## Introduction

Large-granular lymphocytic (LGL) leukemia is a hematologic malignancy caused by cytotoxic T lymphocytes in 85% of cases and by natural killer cells in the remaining cases (1). T-cell LGL leukemia is a rare chronic lymphoproliferative disorder typically characterized by the monoclonal expansion of CD3+ cytotoxic large granular lymphocytes, cytopenia, and splenomegaly (2). Most patients with T-cell LGL leukemia in the Western Hemisphere present with chronic neutropenia of unknown cause, which makes them prone to developing recurrent bacterial infections (3). The possibility of fungal and other pathogen infections should be considered in patients with febrile neutropenia and who are not responding to empirical antimicrobial therapy. Antifungal treatment should be started promptly in these patients based on the fungal diagnosis and treatment guidelines for patients with hematological diseases (4). Here we report a case of T-cell LGL leukemia, in which the patient was repeatedly infected with Aspergillus but was successfully treated.

## Case Presentation

**Figure 1:**
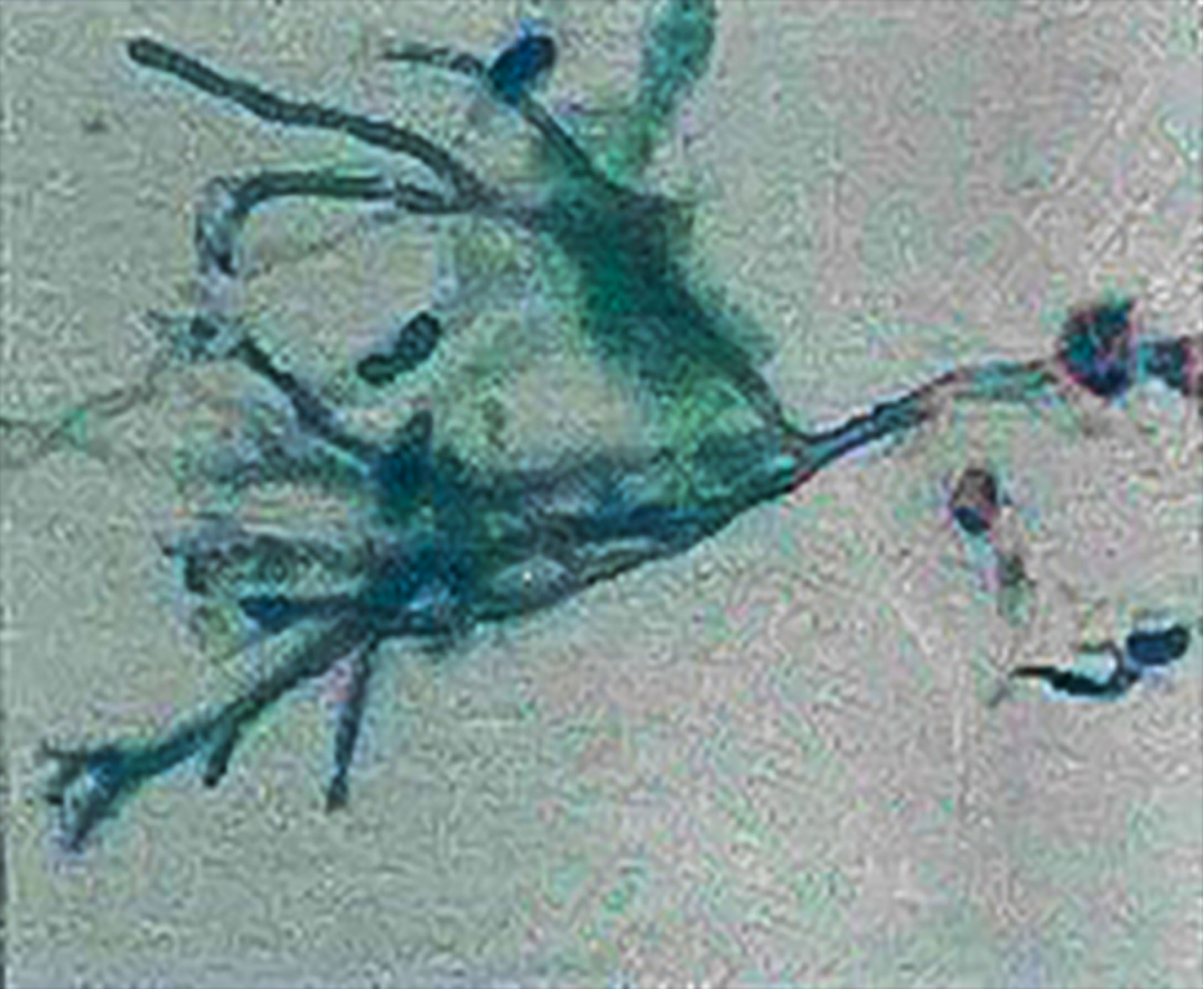
Bronchoscopy liquid-based cytology showed Aspergillus.

A 30-year-old female patient was found to have decreased white blood cells (WBCs) when she saw a doctor for diarrhea in 2015, which did not attract attention. In early April 2017, she developed a fever of more than 38°C, accompanied by a sore throat, and was hospitalized. She had no special medical, family and psychosocial history. The lung fiber bronchoscopy showed abnormal echogenic lesions in the right lower lung basal segment, and bronchoscopy liquid-based cytology showed Aspergillus (**Figure 1**). The diagnoses of agranulocytosis, pulmonary fungal infection, drug-induced rash, hypoproteinemia, and severe anemia were made. The patient still had fevers intermittently after discharge until being re-hospitalized at the end of April 2017. During this hospitalization, she was diagnosed with suspected T-cell LGL leukemia. The patient’s body temperature returned to normal after receiving cyclosporine, methylprednisolone, and voriconazole.

The patient had a relapse in July 2017. Blood tests showed that WBCs were 1.32×10^9/L, neutrophils 0.03×10^9/L, and red blood cells (RBCs) 2.53×10^12/L. A bone marrow pathological biopsy showed a decrease in hematopoietic cells between the trabecular bone marrow. There were many scattered lymphoid cells among the hematopoietic cells, which were suggested to be T cells by immunohistochemical tests. Combined with the immunohistochemical characteristics and particular staining results, the possibility of early involvement of T-cell lymphoma in bone marrow could not be excluded. Bone marrow cytology showed that the proliferation of bone marrow granulocytes was extremely inhibited. The T-cell receptor (TCR) gene rearrangement test in bone marrow was negative for TCR beta (*TCRB*), TCR gamma (*TCRG*), and TCR delta (*TCRD*). The previous T-cell LGL leukemia diagnosis was confirmed. The patient again received cyclosporine, methylprednisolone, and voriconazole for treatment. After discharge, the patient maintained a normal body temperature for more than three and a half years.

**Figure 2:**
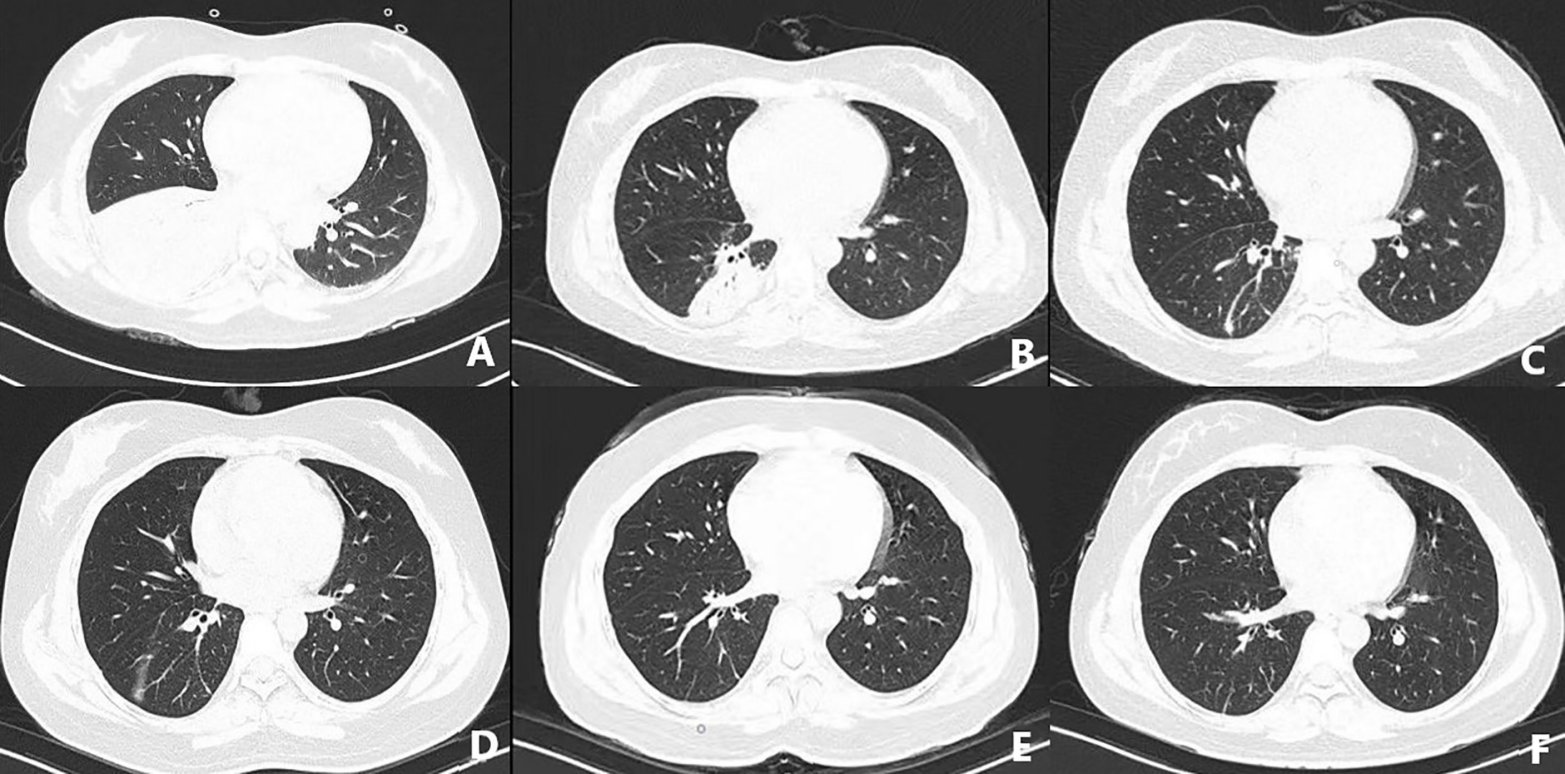
CT-scan of the lungs in the early stage of disease **(A)**, after treatment **(B–D)**, review after one month **(E)**, and review after three months **(F)**.

**Figure 3:**
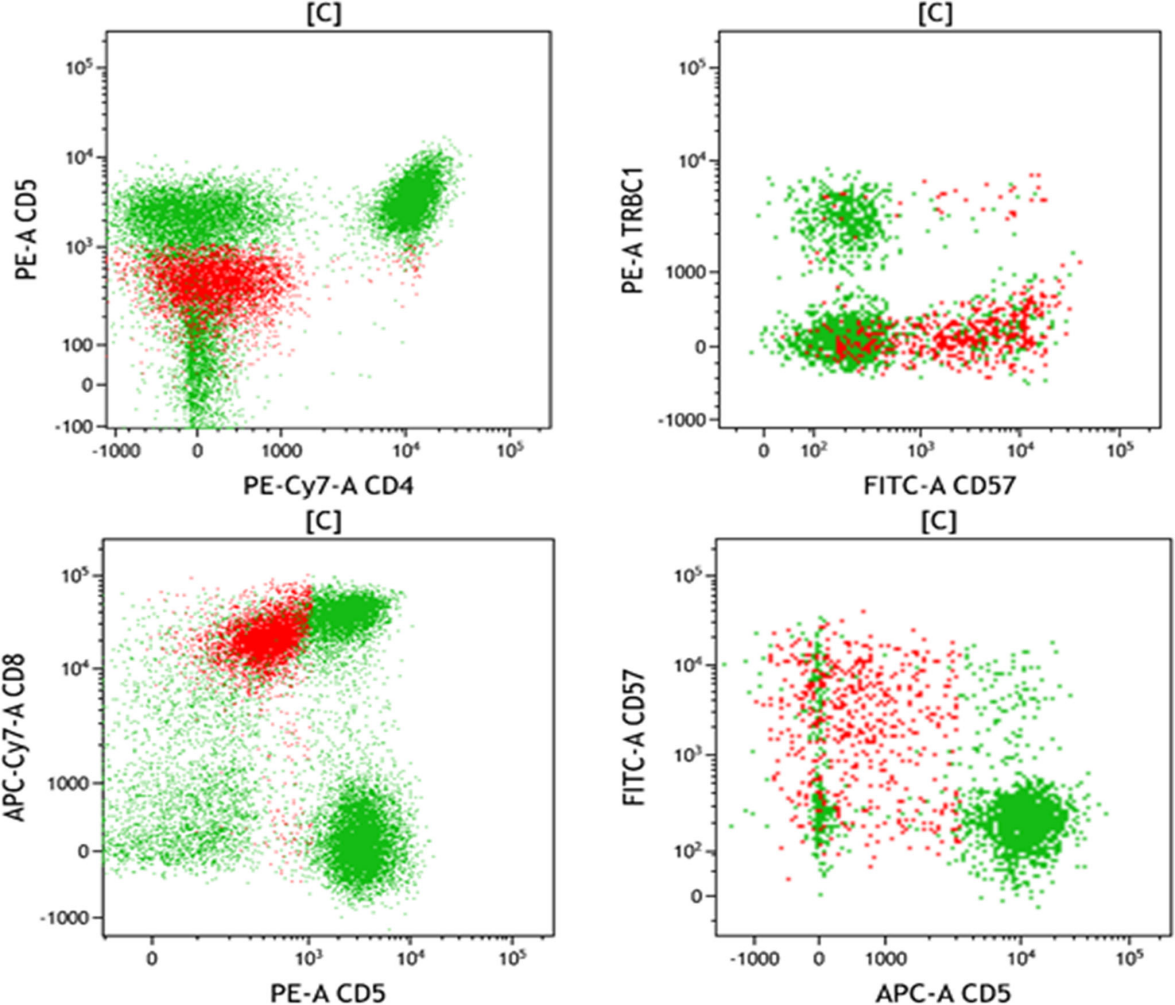
The flow phenotype of the blood cells presented as two-dimensional graphs.

On March 3, 2021, the patient suffered right flank pain and discomfort in the tail vertebra after she stopped taking medications due to intolerable nausea and vomiting. Blood routine showed that WBCs were 0.63×10^9/L, and lung CT showed a mass in the right lower lung (**Figure 2**). On March 4, the patient developed chest tightness, shortness of breath, and malaise, with a fever up to 38°C. Flow cytometry found phenotypes of CD45+, CD3 dim+, CD5-, CD4-, CD8+, CD7+, CD57 p+, CD25-, and CD30-, revealing atypical abnormal T cells (0.8%) (**Figure 3**). TCR test showed positive γδ rearrangement, and metagenomic next-generation sequencing (mNGS) detected Aspergillus. Etiological examinations of the lower respiratory tract specimens were negative, with no fungal growth after seven days of culture. The serum (1,3)-β-D glucan (G-test) and galactomannan (GM-test) were negative. The patient was diagnosed with agranulocytosis and pulmonary fungal infections (Aspergillus). The patient received voriconazole (0.4g q12h followed by 0.2g q12h) and inhaled amphotericin B (5mg bid). The therapeutic drug monitoring (TDM) of voriconazole was performed to adjust voriconazole dosing. On March 9, the patient’s temperature rose to 38.2°C. Also, she received vancomycin, piperacillin/tazobactam, imipenem/cilastatn and linezolid successively for empirical antibacterial therapy.

**Figure 4:**
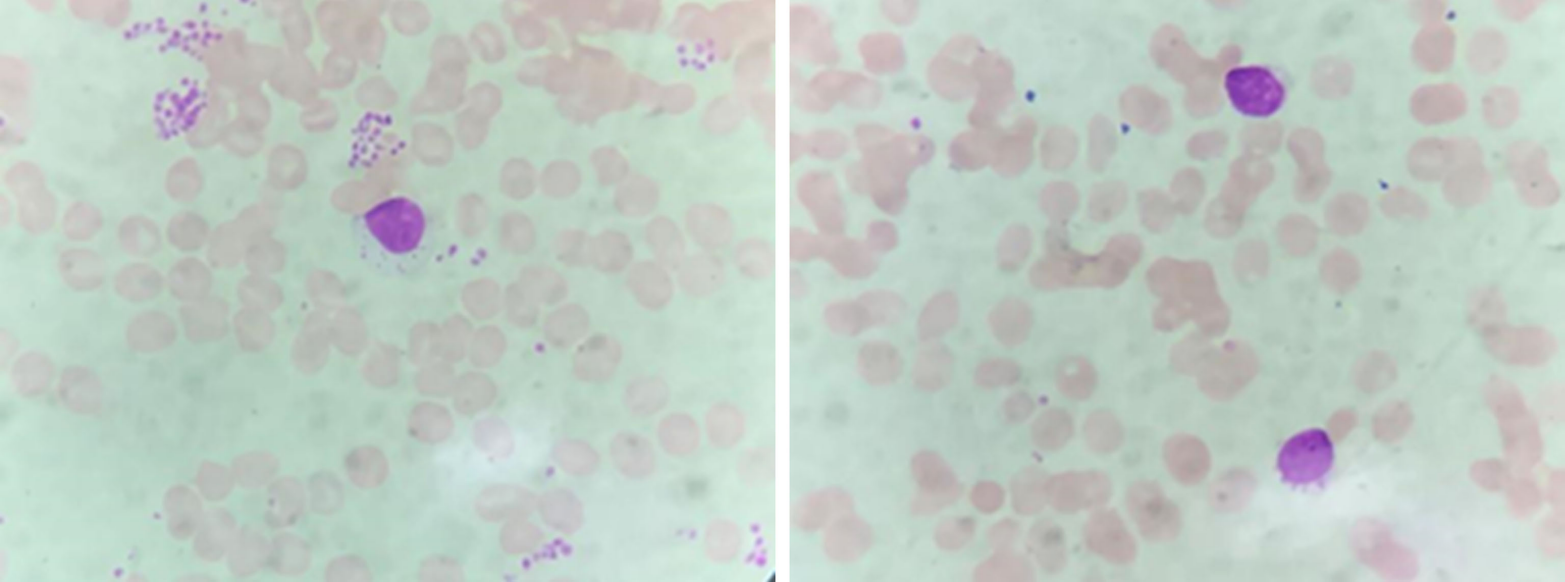
Large granular lymphocytes in bone marrow cytology smear.

**Figure 5:**
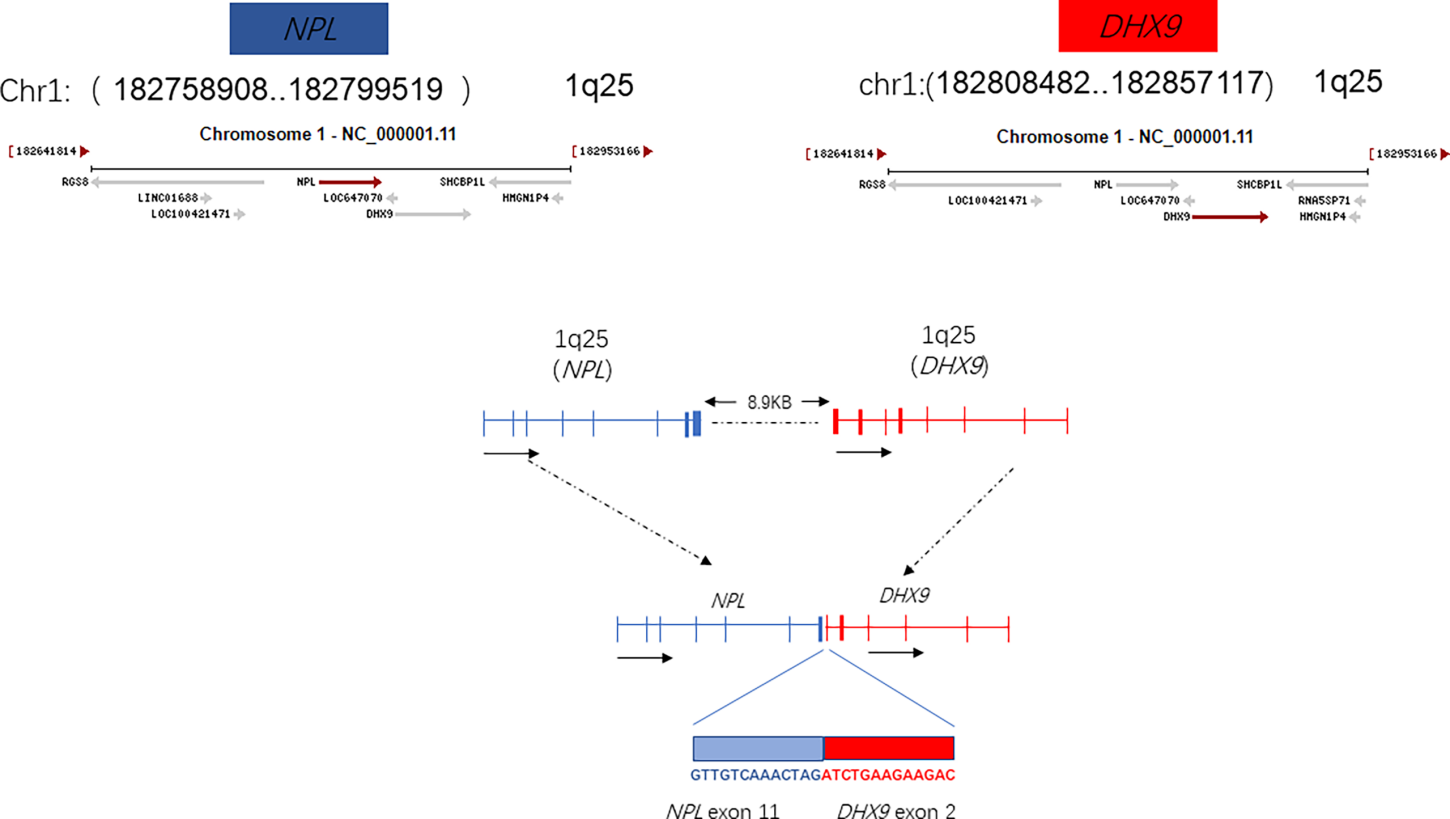
Fusion gene NPL-DHX9 rearrangement through an RNA-sequencing analysis.

The plasma concentration of voriconazole reached 5.393μg/mL on March 17, and the dosage was reduced to 0.15g once a day. Amphotericin B nebulized inhalation was discontinued because of coughing and aggravated pain. The results of the bone marrow smear showed active bone marrow hyperplasia with increased erythrocyte colonies and decreased lymphocyte colonies compared to those of the last time (**Figure 4**). A peripheral blood smear showed an increase in large granular lymphocytes, accounting for 43% of WBCs. Flow cytometry showed that 8.37% of the cells were abnormal mature γδ T cells. The signal transducer and activator of the transcription-3 (STAT3) Y640F mutation and fusion gene NPL-DHX9 rearrangement were confirmed by RNA-sequencing analysis (**Figure 5**). The patient was diagnosed with γ/δ variant T-cell LGL leukemia (NPL-DHX9 positive) on March 22. After assessment, treatment for T-cell LGL leukemia planned to be initiated as soon as possible when pulmonary infection improved.

**Figure 6:**
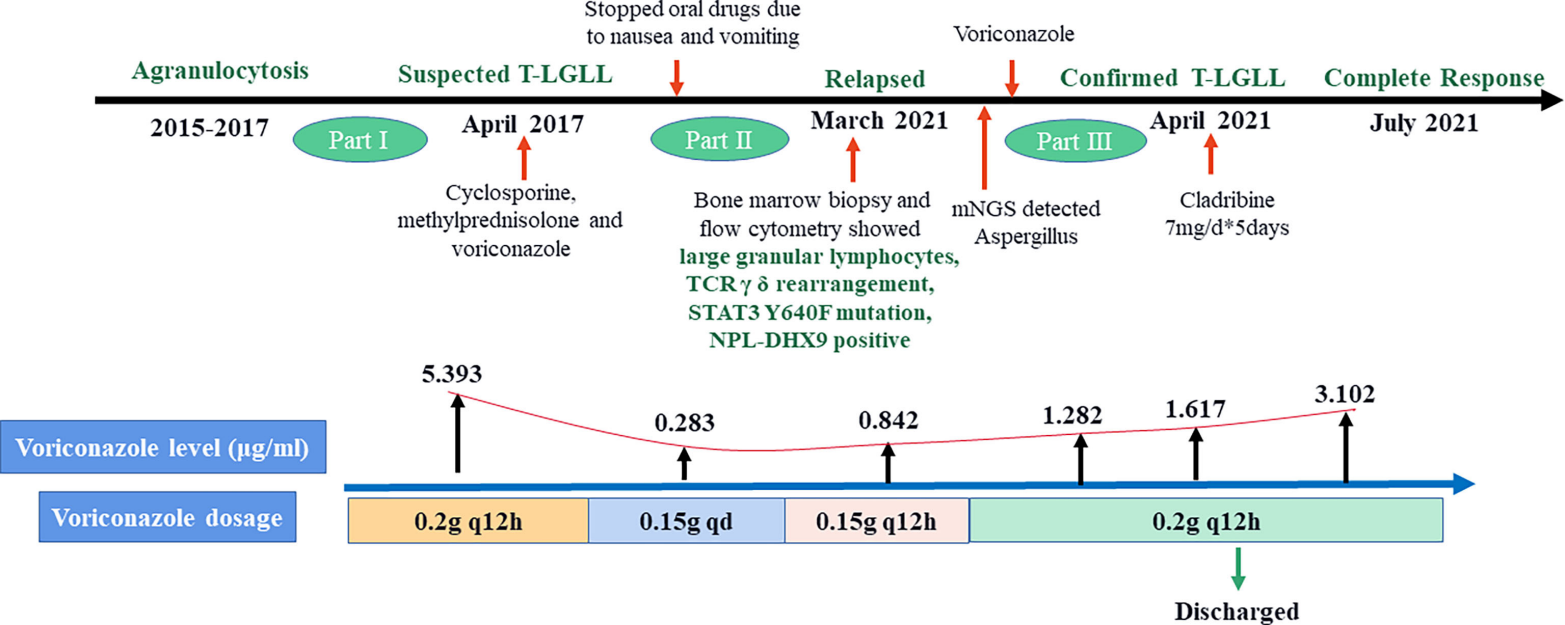
Timeline of disease and treatment periods.

On March 24, the symptoms of chest tightness and shortness of breath mostly disappeared, and the patient was fever-free. When the lung CT on April 8 showed further improvement (**Figure 2**), Cladribine was administrated at 0.14mg/Kg/day daily for 5 days. The patient had no fever for nearly half a month and had no other discomfort, and she was discharged on May 2. Complete response was achieved after four-cycles cladribine treatment. The timeline of the disease and treatments are shown in **Figure 6**.

## Discussion

T-LGLL is a rare and indolent clonal disease of the blood system. The key features of the disease are cytopenia, splenomegaly, and autoimmune manifestations. The median overall survival time upon the emergence of T-LGLL is greater than ten years (5). A diagnosis is established when there is a significant increase of T-cell LGL in the peripheral blood >2×10^9/L (normal, <0.3×10^9/L) or an LGL count between 0.4–2×10^9^/L with related clinical signs and symptoms and hematological features. The colonies of LGL T-cells can be detected with PCR with TCR probes (6). Flow cytometry is used to assess the expression of cell surface markers. Bone marrow aspirate or biopsy is not routinely performed unless it is challenging to make a diagnosis (6). STAT3 is the first molecular marker which is highly specific for LGLL with a mutation frequency of 40% as seen in our patient (7). STAT3 mutations have been rarely detected in other tumor types studied, thus it can be used as molecular markers for LGLL diagnosis and can provide a novel therapeutic target for patients with LGLL.

This patient also harbors a fusion gene of NPL-DHX9 (46.01%), which has never been reported in hematological diseases. The identification of certain activating fusions can contribute to the diagnosis and effective treatment of patients with tumours harbouring these alterations. Although many of noncoding RNAs have been annotated, only a small number of them have been functionally characterized. The Structural domain of N-acetylneuraminate pyruvate lyase (NPL) is still unknown with few genetic studies, sialic acid catabolism by NPL is essential for muscle function in a recent study (8). DEAH (Asp-Glu-Ala-His) box helicase 9 (DHX9), an RNA helicase which regulates the expression of genes at the transcriptional and translational levels. DHX9 is involved in multiple gene regulatory steps, including transcription, translation, and miRNA-mediated control, DNA replication, and maintenance of genome stability. DHX9 has been implicated in tumor cell maintenance and drug response (9). DHX9 knockdown increased the protein and mRNA levels of CDK6, which consequently resulting in hepatocellular carcinoma development (10). High expression of DHX9 promotes the growth and metastasis of hepatocellular carcinoma (11). This article describes fusion gene NPL-DHX9 rearrangement confirmed by RNA-sequencing analysis: 1) The 5’ end gene is NPL which contains 36-205 amino acids with domain unknown; 2) The 3’-end gene is DHX9 which contains 1-166 amino acids, retains the DRBM1 domain, but as an RNA helicase gene, the helicase functional domain is not retained. From the perspective of fusion structure alone, it does not play an important function or the function is not clear for the time being. The NPL-DHX9 fusion gene was detected and the protein was expressed in our patient, however, whether the fusion is relevant to the diagnosis of the disease and to the treatment or prognosis cannot be predicted precisely at present and in-depth analysis such as immunohistochemical testing is required.

There are no established clinical guidelines for treating T-cell LGL leukemia, Immunosuppressive agents are frequently used in T-LGLL, and methotrexate (MTX) and cyclophosphamide remain first-line therapies. Loughran et al. studied the correlation between the STAT3 gene and the efficacy of immunosuppressive agents. The results showed that patients with mutations responded better to MTX, especially in those with the STAT3 Y640F mutation genotype (12). Zhu et al. found that αβ T-LGLL and γδ T-LGLL have same pathogenesis and similar clinical manifestations. Anti-human T-cell immunoglobulin (ATG) or high-dose cyclophosphamide was recommended as the second-line treatment regimen for γδ T-cell LGL leukemia (13). Purine nucleoside analogues (PNA) are cytotoxic agents highly active in the treatment of indolent lymphoid malignancies and should be considered in patients with widespread disease. Fludarabine and pentostatin were the most studied purine nucleoside analogs (5).

Few case reports or clinical studies of using cladribine in treating large granular T-cell leukemia were able to identified. A study probed synergistic effects in induction of apoptosis in leukemia NK cells using low doses of cladribine in combination with vorinostat *in vitro*, which suggested that it may be a promising therapeutic strategy for NK-LGL leukemia (14). An 84-year-old man diagnosed with T-LGLL and smoldering MM initially received cladribine, then methotrexate, without improvement. The patient finally treated with cyclophosphamide (100 mg daily for 1 year) and achieved complete remission (15). A 50-year-old man with T-LGLL, refractory to prednisone, methotrexate, cyclosporine, and G-CSF who received two cycles of cladribine at 0.1 mg/kg for 7 days and achieved the rapid CR (16). Targeted therapies such as alemtuzumab are promising and may be used in the future, particularly in relapsed or refractory patients. Granulocyte colony-stimulating factor (G-CSF) as a single agent has poor efficacy in treating T-LGLL. Stem cell transplant is presently not suggested unless patients are not responding to other treatment options (5). Unfortunately, 97% of drugs tested in early clinical stages failed usually because of dose-limiting toxicities and unsatisfactory efficacies (17). Large-sample clinical trials to detect significant differences between treatment groups are challenging, and multi-center studies are necessary (6). In our case, the second-line treatment drug, cladribine, was selected due to the patient’s past drug intolerance to long-term use of immunosuppressants. The patient achieved complete remission after four courses of cladribine chemotherapy, without intolerance or recurrence.

Long-term use of immunosuppressants combined with agranulocytosis is a double-edged sword; both can lead to serious infections. The main goal of LGL leukemia treatment is to relieve symptoms, especially to control infections caused by neutropenia (18). The CAESAR study showed that among patients with hematological malignancies who received chemotherapy, the total incidence of confirmed and clinically diagnosed invasive fungal disease (IFD) was 2.1% (19). Patients with T-cell LGL leukemia are more vulnerable to severe opportunistic fungal infections, while neutropenia is an independent risk factor. Common pathogens of IFD in immunodeficiency patients are Aspergillus and Candida (20). In addition, IFD in immunocompromised patients are associated with higher mortality and treatment costs (21). Early implementation of antifungal therapy is the standard approach for managing patients of hematological malignancies with a suspected fungal infection, especially during the neutropenia phase (21). The 2016 American Society of Infectious Diseases (IDSA) guidelines recommend voriconazole as the first-line choice for the treatment and prevention of IPA (22). Repeated monitoring of the plasma concentration of voriconazole is recommended when adjusting the voriconazole dose, the occurrence of adverse events, poor efficacy, and adding or stopping drugs that can affect the pharmacokinetics of voriconazole (23).

## Conclusion

T-cell LGL leukemia is a rare hematological condition involving TCR rearrangement and functional T-cell deficiency. Patients are more vulnerable to severe opportunistic fungal infection, while neutropenia is an independent risk factor. We report a patient diagnosed with T-cell LGL leukemia with a rare γ/δ type and STAT3 mutation (Y640F). The fusion gene NPL-DHX9 rearrangement was confirmed by RNA-sequencing analysis, which has never been reported in hematological diseases. The relevance of this novel fusion gene to the pathogenesis, diagonosis, prognosis, and treatment of LGL leukemia may be further evaluated through in-depth analyses which are in progress. Complete response was achieved after 4-course cladribine treatments. Aspergillus infection was confirmed and ultimately successfully treated with voriconazole. Our case illustrates the diagnostic and therapy key points of invasive aspergillosis in patients with T-cell LGL leukemia.

## Data Availability Statement

The original contributions presented in the study are included in the article/supplementary material. Further inquiries can be directed to the corresponding authors.

## Ethics Statement

Written informed consent was obtained from the individual(s) for the publication of any potentially identifiable images or data included in this article.

## Author Contributions

QH and YL searched relevant references and drafted the manuscript. YZ and SS provided insights for text and figures. HW revised the manuscript. SD and ZJ originated the work, made comments, and revised the manuscript. All authors read and approved the final manuscript.

## Funding

The study was supported by grants from Natural Science Foundation of Hunan Province, China(2018JJ2660). The funders had no role in study design, data collection and analysis, decision to publish, or preparation of the manuscript.

## Conflict of Interest

The authors declare that the research was conducted in the absence of any commercial or financial relationships that could be construed as a potential conflict of interest.

## Publisher’s Note

All claims expressed in this article are solely those of the authors and do not necessarily represent those of their affiliated organizations, or those of the publisher, the editors and the reviewers. Any product that may be evaluated in this article, or claim that may be made by its manufacturer, is not guaranteed or endorsed by the publisher.
